# The Mexican Cycle of Suicide: A National Analysis of Seasonality, 2000-2013

**DOI:** 10.1371/journal.pone.0146495

**Published:** 2016-01-11

**Authors:** Julián Alfredo Fernández-Niño, Claudia Iveth Astudillo-García, Ietza Bojorquez-Chapela, Evangelina Morales-Carmona, Airain Alejandra Montoya-Rodriguez, Lina Sofia Palacio-Mejia

**Affiliations:** 1 Information Center for Decisions in Public Health (CENIDSP), National Institute of Public Health of Mexico, Cuernavaca, Mexico; 2 Mexico School of Public Health, Cuernavaca, Mexico; 3 Department of Population Studies, Colegio de la Frontera Norte, Tijuana, Mexico; 4 Department of Reproductive Health, Population Health Research Center, National Institute of Public Health of Mexico, Cuernavaca, Mexico; University of Geneva, SWITZERLAND

## Abstract

**Introduction:**

Suicide is a complex and multifactorial phenomenon with growing importance to public health. An increase in its occurrence has been observed in Mexico over the past 10 years. The present article analyzes the secular trend in suicide at the national level between the years 2000 and 2013.

**Materials and Methods:**

All suicides during the study period (n = 64,298, of which 82.11% were men) were characterized using a spectral decomposition of the time series and a wavelet analysis to evaluate the effect of seasonal changes, type of area (urban versus rural) and sex.

**Results:**

A seasonal pattern was observed with statistically significant cycles every 12 months, where peaks were identified in May but only for men in urban zones as of the year 2007. In addition, specific days of the year were found to have a higher frequency of suicides, which coincided with holidays (New Year, Mother’s Day, Mexican Independence Day and Christmas).

**Conclusion:**

A wavelet analysis can be used to decompose complex time series. To the best of our knowledge, this is the first application of this technique to the study of suicides in developing countries. This analysis enabled identifying a seasonal pattern among urban men in Mexico. The identification of seasonal patterns can help to create primary prevention strategies, increase the dissemination of crisis intervention strategies and promote mental health. These strategies could be emphasized during specific periods of the year and directed towards profiles with a higher risk.

## Introduction

Worldwide, one suicide occurs every 40 seconds and an estimated 20 suicides are attempted for every one completed [1]. This phenomenon is present in all cultures and regions around the world as well as over nearly the entire course of people’s lives. According to data from the latest report by the World Health Organization (WHO) [1], an estimated 804,000 suicides occurred in 2012 worldwide, with an age-standardized rate of 11.4 per 100,000 inhabitants (15.0 for men and 8.0 for women). By the year 2020, suicide will contribute to more than 2% of the global burden of disease [2]. And in Latin America, an increasing trend in the rate of suicides has been observed over the past decade [3, 4]. In the case of Mexico, the suicide rate for both sexes was 1.5 per 100,000 inhabitants in 1959 [5], while it was 2.89 in 1994 [6] and 4.8 in 2008 [5]. When comparing age-standardized rates in 2000 (3.6 per 100,000 inhabitants) and in 2012 (4.2 per 100,000 inhabitants), an increase of 16.6% is observed [1].

The WHO has reported that the main risk factors for suicide are: mental disorders (primarily depression and those caused by substance abuse), grief, sense of loss and disability, in addition to stressful cultural and social environments, especially those with violence or discrimination [1]. Therefore, it has been suggested that the completion of suicide depends on both the social environment and its interaction with individual personality traits [2].

This relationship between suicides and the effects of the physical external environment has been recognized in documents dating back to the 19^th^ century [7], including seasonality, which refers to the fact that suicides have a monthly pattern which tends to repeat yearly [8]. The seasonality of suicide has been studied based on two broad perspectives, biological and sociological. Works by Morselli (1881) [7–9] have influenced the biological perspective, including his analysis of 18 European countries in which he reported that 88% of suicides occurred during summer (June-August). This author therefore proposed that weather-related factors (especially heat) increase the sensitivity of the nervous systems which would explain the increase in suicides during spring and early summer [10]. Studying suicide from a sociological perspective, Durkheim (1897) [11] proposed an opposing theory that increased social activity during spring and summer creates more social stress, and this could lead to suicidal behavior when unable to psychologically cope with the frustration caused by failed social interactions, especially for people with depression [9, 12, 13]. More recent studies of suicides have shown seasonal patterns in different regions and countries, such as the review by Roehner of data from France, Ireland, South Korea and Switzerland [8]. A study by Petridou, *et al*. analyzed seasonal trends in suicides in 20 countries belonging to the Organization for Economic Co-operation and Development (OECD) for the period 1995 to 2012, found that the suicide rates peaked in June in the northern hemisphere and in December in the southern hemisphere[14]. In terms of Latin America, similar explorations have been conducted only in Brazil [15, 16] and Chile [17].

Several techniques have been used to study seasonality, including time series analyses [18, 19], variance analyses [17, 20, 21] and multivariate Poisson models [22, 23]. Nevertheless, according to Cazelles *et al* [24], these classic methods are more useful for relatively stationary time series than for long epidemiological time series, given that they are usually noisy, complex and non-stationary. Therefore, a wavelet analysis is a good alternative to study phenomena such as suicide. Using this technique, the characteristics of the time series can be adequately determined and the effects can be separated by time periods corresponding to a calendar year [24]. This analytical technique can improve the characterization of the seasonality of suicide as well as increase knowledge about the processes leading up to it and the potential influences of temporary exogenous changes. While this technique has been used in other countries to analyze suicides [25], this is the first investigation of this type in Mexico. Therefore, the aim of the present analysis is to explore the seasonal behavior of suicide in Mexico using spectral decompensation and extensively characterize all the cases occurring at the national level between the years 2000 and 2013.

## Material and Methods

### Ethical Statement

This study is a secondary data analysis of the national vital statistics system of the National Institute of Statistics and Geography (INEGI; Spanish acronym) and the Statistical and Epidemiological System of Deaths (Sistema Estadístico y Epidemiológico de Defunciones(SEED)), of the General Department of Health Information (Dirección General de Información en Salud (DGIS)). The data for the analysis were requested and obtained from the survey’s public repository hosted at the National Institute of Statistics and Geography. This repository has the data already deidentified; thus, it is not possible to trace any of the data to the actual individual. In accordance with the Internal Regulation of the Research Ethics Committee of the National Institute of Public Health, this secondary analysis was considered exempt from approval.

### Source of Data

National level data about suicides registered in Mexico were obtained for the years 2000 to 2013. The data were directly extracted from death-related statistics stored in the national vital statistics system of the National Institute of Statistics and Geography’s (INEGI; Spanish acronym) [26]. This database contains basic sociodemographic information about the deceased (sex, age, education, occupation, health insurance or clinic affiliation), as well as the date and place of the event and the date and place it was registered. These statistics were collected from the administrative records from various public offices. Information was inputted from certificates and statistics registries obtained from a total of 6,743 sources of information throughout the country, including documents from civil registries, family, civil and mixed court proceedings and public ministry agencies, which provide monthly information. In addition, a necropsy was performed to confirm the cause of death for each case involving a suspected accident, suicide or homicide, or a work-related death. Other details about suicide, such as the method used, was obtained from the Statistical and Epidemiological System of Deaths (Sistema Estadístico y Epidemiológico de Defunciones (SEED)), of the General Department of Health Information (Dirección General de Información en Salud (DGIS)) for the same time period. This is a subsystem which compiles national level mortality information from the same primary sources mentioned above. (More information about this subsystem can be found at http://www.dgis.salud.gob.mx/contenidos/sinais/s_seed.html).

Rates were calculated by sex based on population projections by the National Population Council (CONAPO, Spanish acronym), according to population censuses from 2005 and 2010 recorded by municipality, age and sex [27].

### Statistical Analysis

#### Analysis descriptive

A descriptive analysis of the main characteristics of suicide cases was performed according to sex. The qualitative variables were summarized using proportions and the quantitative variables using the respective interquartile ranges. Annual rates were calculated by sex and a trend over the study period was observed at the national level.

#### Analysis univariate time series

The series of the monthly suicides from 2000 to 2013 were analyzed for men and women in Mexico. A linear trend was discarded in the monthly time series given that the time´s coefficient in a standard least squares regression was not statistically significant (p>0.10).

Later, the autocorrelation was verified for both sexes using partial autocorrelation graphs and the Wallis test [28]. This consisted of adapting the Durbin-Watson statistic [29] for models with seasonality, in which a disturbance in a concrete observation is not only related to the disturbance corresponding to the immediate previous period but also to the same period (month) from the previous year. Finally, the seasonality of the series was verified using the Dickey-Fuller test [30] to test the null hypothesis of the existence of a unit root in an autoregressive model versus the alternative that more than one trend exists in the time series.

These tests were previously performed as part of a standard exploratory time series analysis and to obtain a “traditional” reference point for the subsequent wavelet analysis. In general terms, the main purpose was to explore if the stationary trend had a unit root in the time series for men and for women, as well as the order of autocorrelation of the observations.

#### Wavelet analysis

A wavelet analysis is a local spectral decomposition of a time series into two components: time (t) and frequency-period (f) [24]. The justification for using this technique was described previously in a recent paper [25].

Statistically, this technique can be particularly useful when the series does not maintain its mathematical properties over time. In epidemiological terms, this can occur especially when changes in a phenomenon are seasonal or when complex or noise patterns exist, as typically happens with very long time series [31]. This technique has been widely used in epidemiology to analyze the behavior of infectious diseases, and particularly to analyze their relationships with climate-related variables [24].

In brief, the wavelet transform decomposes a signal using functions (wavelets). This decomposition produces a good time-frequency localization of the intensity of the signal (the intensity in an epidemiological time series refers to the number of cases). In brief, a wavelet function is derived from a signal *X*(t) (a time series), defined as:

$$W_{x}\;\left(\;\alpha ,\tau \;\right)=\;\frac{1}{\sqrt{\alpha }}\;\int_{-\infty }^{\infty }X\left(t\right)\Psi *\left(\frac{t-\;\tau }{\alpha }\right)\text{d}t\;=\;\int_{-\infty }^{\infty }X\left(t\right)\Psi *\left(t\right)\text{ d}t$$

where x(*t*) represents the time series for each value of *x* (the intensity) at each time *t*, α is the window (resolution) within which the change is examined at each two-dimensional coordinate corresponding to calendar time (*t*) and period (τ), and where Ѱ is the wavelet function. Thus the wavelet transform mainly appears as a linear filter with its response function given by the wavelet function. The appropriate wavelet function needs to be selected for the wavelet analysis. These functions are generally characterized by low oscillations with good frequency and poor time resolution or fast oscillations with good time resolution but a lower frequency resolution [24]. Even though discrete functions can be more quickly implemented in practice, the present study did not use them because they are affected by the quantity of data. In addition, orthogonal functions and redundant decompositions are beyond the scope of the present analysis and their implementation and interpretation involve a high degree of complexity. Therefore, a continuous wavelet function was chosen for the present study.

The Morlet function is one of the most common continuous wavelet functions used in epidemiology [32–34]. This was applied by the present study since it makes it possible to create graphs that are easy to interpret and provides a simple way to analyze the time and period components of time series, which is more consistent with the objective of the present analysis.

The wavelet analysis is interpreted based on a reading of two-dimensional graphs, in which the *y*-axis corresponds to the period (τ) and the *x*-axis to time (*t*). The period (τ) refers to the cycle, that is, how often the peaks repeat. Time (*t*) refers to calendar time. Thus, the value of the wavelet function is estimated for each coordinate (*t*, *τ*). The graph displays different colors according to the intensity of the wavelet function at each point (*t*, *τ*), where warmer colors represent higher intensities. The areas corresponding to the period-time during which the infection had the highest intensity can thereby be viewed simultaneously. In addition, the areas that are statistically significant under the null hypothesis are encircled by a dark line.

The effects should be analyzed within a cone of influence to eliminate boundary effects, since the analysis presumes a circular convolution in the time series in which the last point is connected to the first. Although the mathematical details of this model are beyond the scope of the work herein, which is an empirical application, they can be found in the available literature [25, 34].

Before performing the analysis, a square root transform was applied to the number of monthly suicide cases in order to manage the variability in the series over time and to normalize the amplitude, as has been done by various previous studies that use this technique [32, 35–37]. Lastly, the statistical significance was not evaluated based on the widely used null hypothesis that the variability in the time series is the same as the variability expected from a purely random process [24, 38]. Instead, our null hypothesis was based on a surrogate series generated by a first-order autoregressive process [38], given that the autocorrelation pattern in the univariate analysis of the series was very high for a first-order (as will be shown in the results).

The significant portions of the graphs are indicated by shaded areas, at an α of 0.05. All the analyses were performed with the R package "biwavelet", version 3.2.0 [39] and STATA version 12.0 [40].

Technical note: The authors state that all the analytical procedures are fully reported in the methodology section of this article. In addition, the description of the results is exhaustive and no data was excluded. Thus the findings can be replicated by following the procedures described in the article. This also implies that no analyses were excluded from the present report, including those that were negative. Finally, the period 2000 to 2007 was selected because it was the period with the information available.

## Results

**Fig 1:**
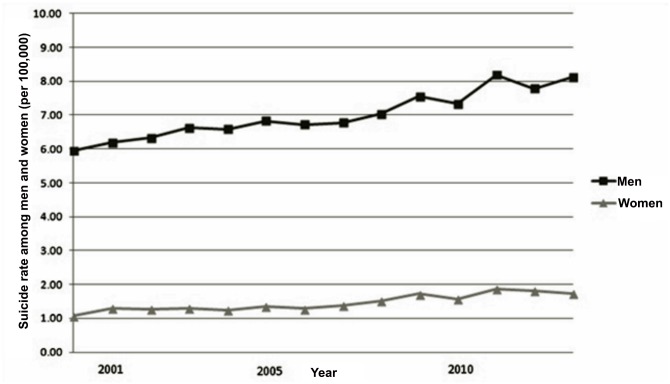
Annual Trend in the National Suicide Rate in Mexico, per sex, 2000–2013.

The sample was composed of a total of 64,298 suicide cases registered in Mexico during the period 2000 to 2013. Table 1 summarizes the main characteristics of the cases studied. Of the total, 52,797 were men (82.11%) and 11,485 were women (17.86%). The suicide rate for men increased from 5.95 in 2000 to 8.12 in the year 2013 (per 100,000 inhabitants), and from 1.06 to 1.73 for women during the same period. Fig 1 presents the trend in the national annual suicide rates per sex. In the national series, a general increasing trend is observed over the last 13 years of the study period, especially as of 2007.

**Table 1:** Characterization of Historical Suicide Cases in Mexico by sex, 2010–2013.*

Variable	Total**	Men	Women
	(n = 64,298)	(n = 52,797)	(n = 11,485)
**Age**			
Mean (SD)	35.63(17.82)	36.70(17.98)	30.69(16.05)
Median (IR)	31(22–45)	32(23–46)	26(18–40)
**Civil Status**			
Single	41.88	41.06	45.73
Married or Free Union	37.66	38.23	35.05
Divorced or Separated	11.85	12.12	10.61
Widowed	4.93	4.85	5.42
**Schooling**			
None	6.65	6.89	5.57
Elementary	44.13	45.46	38.11
Secondary	33.07	32.13	37.44
Bachelor’s or higher	15.14	14.52	17.99
**Occupation**			
Directors and Upper Management	0.35	0.39	0.16
Professionals, salaried workers and technicians	26.34	18.81	61.08
Assistant workers	11.13	11.44	9.74
Business persons	7.03	7.82	3.42
Personal services and security	4.07	4.58	1.71
Farm or livestock workers	16.1	19.39	0.96
Machine operators and drivers	19.91	23.79	2.05
Unemployed	5.01	3.49	12.05
**Health care affiliation**			
None	46.35	47.42	41.46
Social Security or other	26.12	25.41	29.41
Subsidized social protection	7.45	6.76	10.63
Unspecified	20.08	20.41	18.49
**Place of occurrence**			
Home	72.31	67.5	63.93
Public places	8.98	3.04	4.1
Farm (ranch or rural lot)	2.71	2.29	2.84
Other	5.08	6.76	10.63
No data	10.93	20.41	18.49
**Area**			
Urban	78.36	78.34	78.41
Rural	21.64	21.66	21.59

* Proportions of the total and within each sex are presented (except for age which is described as a quantitative variable).

IR = interquartile range.

** The total does not represent the exact sum due to a lack of data pertaining to sex.

In terms of age, the mean was 35.63 years with a standard deviation (SD) of 17.82; a mean of 36.70 (SD 17.98) was determined for men and 30.69 (SD 16.05) for women. With regard to the other sociodemographic characteristics, the percentage (41.88%) who were single at the time they completed suicide is most notable (41.06% for men and 45.73% for women), followed by the category corresponding to married or in a free union, for both groups (38.23 and 35.05, respectively). Similar distributions in education are also observed, the most frequent being elementary school (incomplete or complete) with 44.13% of the total (45.46% for men and 38.11% for women).

In terms of the occupation of the subjects, a statistically significant difference in the type of work activity at the time of death was found between men and women (p<0.01). Machine operators and drivers was the most frequent category for men (23.79%) and salaried work was most frequent for women, representing more than half of the cases registered (61.08%).

For both men and women, a large proportion of the total cases had no access to institutional health services (46.35%). The home was the location where most of the suicides occurred (72% of the total), and the majority of the suicides registered occurred in urban areas (78.36%). Lastly, in terms of the mechanisms used, 77.44% were by strangulation, asphyxiation or hanging, 13.02% by firearms, 7.68% poisoning (gas, pesticides, chemicals, medication and poisons), 0.87% cutting, 0.45% jumping from heights and the remaining 4.22% by other mechanisms (including jumping out of a moving vehicle, setting oneself on fire, etc.).

When exploring seasonality by month in which the suicides were completed, May had the highest frequency for the total period, with 6,108 cases (9.51%); followed by August with 5,678 registered cases (8.84%); the month with the fewest cases was February, with 4,754 (7.4%). It is noteworthy that May was the month with the most suicides (64.29%) in 9 of the 13 years studied, and was the second highest month in 3 of the 4 years when it was not in first place (Table 2). The same pattern is seen by sex, in which the highest percentage of suicides occurred in May for both men (9.49%) and women (9.60%), while February had the least number of cases for men (7.43%) as well as for women (7.27%)

**Table 2:** Description of the three months with the highest frequency of suicides per year, 2000–2013.

	First place (n)*	Second place (n)	Third place (n)
**2000**	May (327)	April (303)	August (302)
**2001**	April (359)	May (352)	June, December (327)
**2002**	December (345)	August (342)	March (339)
**2003**	May (403)	July (372)	March (369)
**2004**	May (416)	September (379)	June (353)
**2005**	May (431)	September (395)	August (389)
**2006**	May (421)	July (394)	August (382)
**2007**	March (421)	May (415)	June (392)
**2008**	May (504)	August (428)	July (400)
**2009**	May (482)	June, August (481)	July (478)
**2010**	May (467)	June (463)	July (461)
**2011**	August (528)	March (513)	May (511)
**2012**	May (519)	June (485)	April (484)
**2013**	June (550)	May (542)	August (535)

*Statistically significant differences were found between the medians in May and the medians of the other months for all the years. (Kruskal-Wallis, p<0.01).

**Fig 2:**
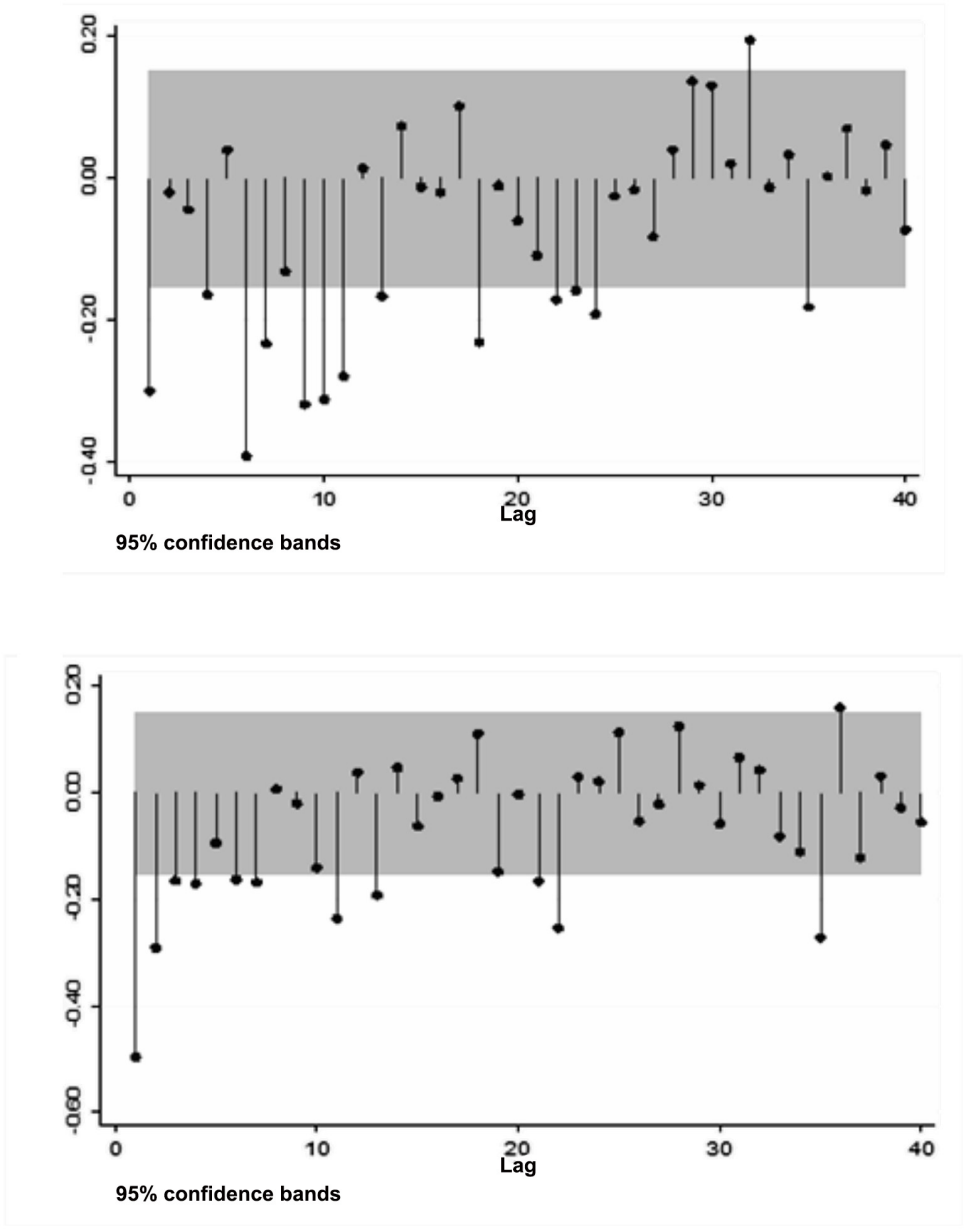
Partial Autocorrelations for the Time Series for Men (above) and Women (below) 2000–2013.

A univariate analysis of the series was performed before the wavelet analysis in order to explore if the trend had a unit root in both series and determine the autocorrelation. The autocorrelation tests using the Wallis comparison showed a significant autocorrelation within each time series (for men as well as for women) (*p*<0.001). In both cases, the Dickey-Fuller test supports the null hypothesis of a single trend, with *p* = 0.833 for the series corresponding to men and *p* = 0.4962 for the series corresponding to women (Fig 2). In other words, this test makes it possible to simply suggest that a single seasonality exists in the time series for men as well as the one for women, which continues throughout the years included in the time series. On the other hand, the most significant partial autocorrelation had a lag of 1 (equal to one month) for both sexes (*r*>0.30, *p*<0.01) and therefore represents the null hypothesis of the wavelet.

**Fig 3:**
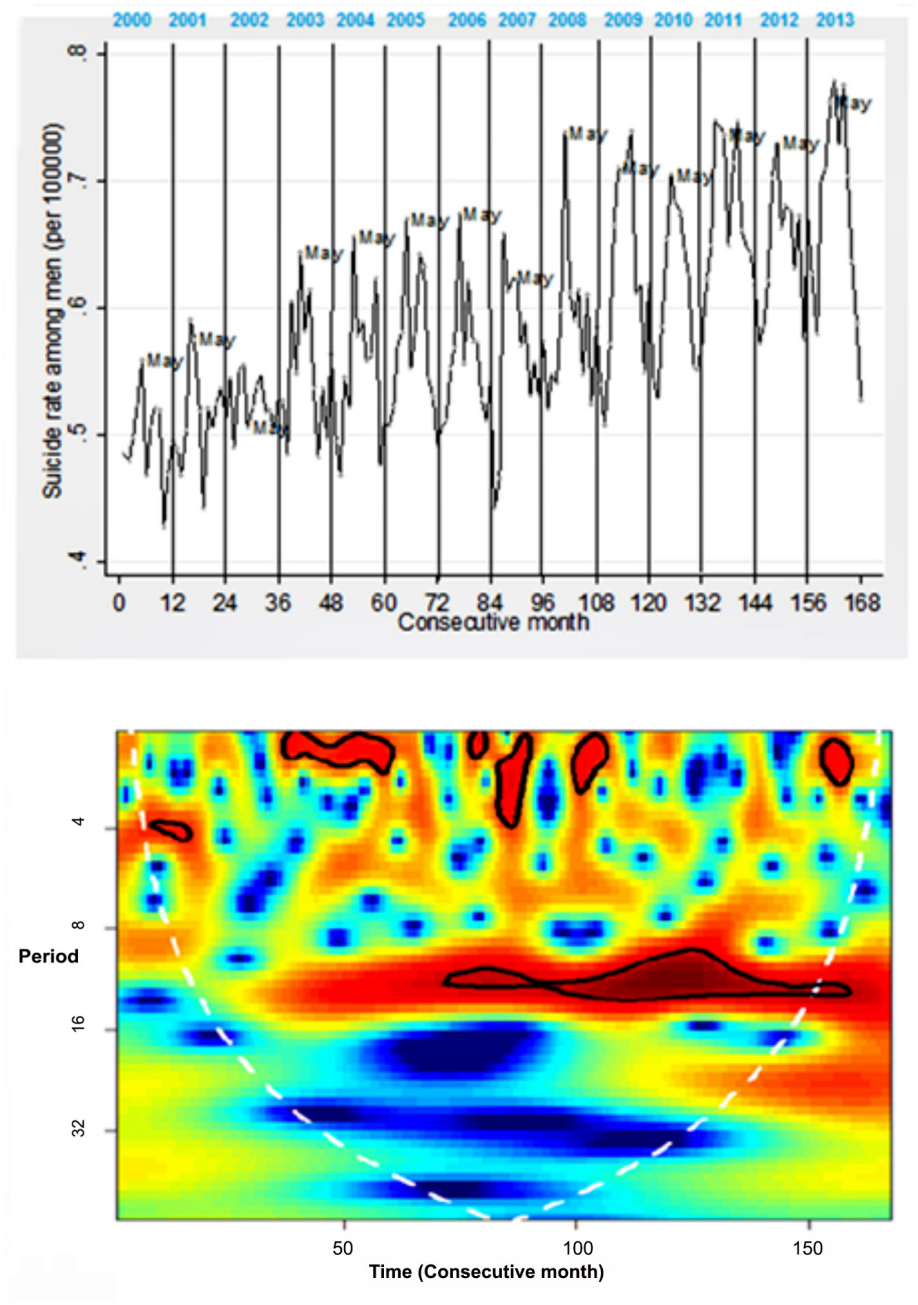
Time Series for National Suicides for Men in Mexico and the Respective Wavelet Power Spectrum, 2000–2013*

**Fig 4:**
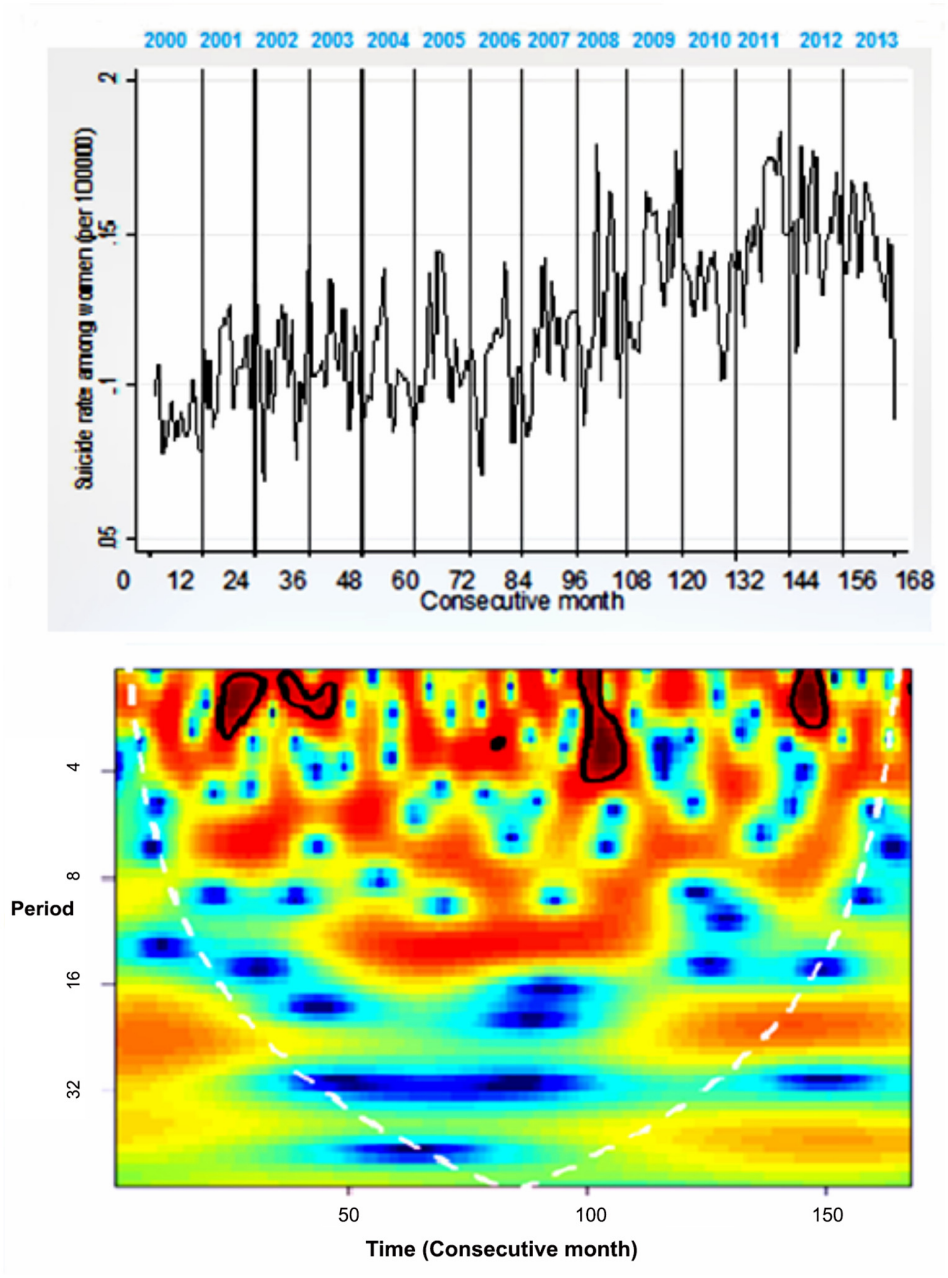
Time Series for National Suicides for Women in Mexico and the Respective Wavelet Power Spectrum, 2000–2013

Regarding wavelet analysis Fig 3 presents the monthly time series for the national suicide rate for men from 2000 to 2013, with its respective wavelet power spectrum. Fig 4 presents the same graphs for women. A consistent seasonal trend beginning in the year 2007 was observed, but only for men. It is important to note that 2007 was the year in which an apparent increase in suicides occurred in Mexico (Fig 1). Peaks were found around the month of May (in the upper part of Fig 3), an effect in the time series which was verified by the spectral analysis.

*The calendar time and period are in months. In the case of the calendar time, the months are consecutive, where 1 is the first month of the year 2000. Warmer colors signify higher intensity, areas encircled by a black line represent statistically significant effects (alpha of 0.05). The white dotted line is the cone of influence and the area inside this cone is not impacted by the boundary effect from the circular convolution of the series.

**Fig 5:**
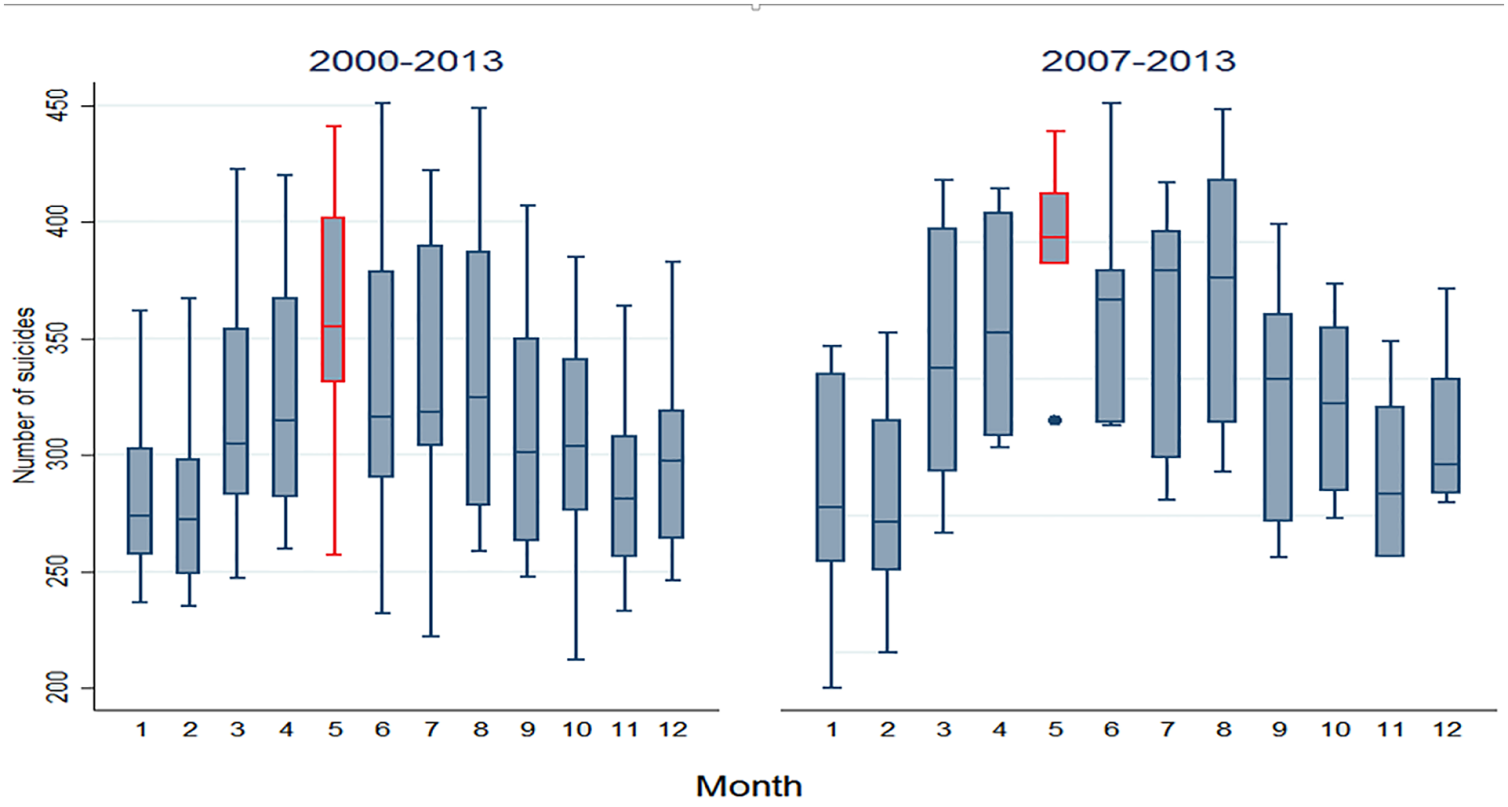
Distribution of the number of suicides among men per month in Mexico from 2000 to 2013 and from 2007 to 2013.

More specifically, the power spectrum for the men’s series (bottom of Fig 3) shows a seasonality every 10 to 12 months, beginning in the year 2007 and continuing until 2013. This seasonality was verified as located in May, when viewed months with higher suicides for each year (Table 2).This power was statistically significant at a level of 0.05, which results in rejecting the null hypothesis that the observed variability in the time series is the same as the variability expected from the random process generated by a first-order autoregressive process [38]. Unlike the men’s series, the series corresponding to the women (bottom of Fig 4) does not present seasonal consistency. To return this finding to its original scale (number of cases), Fig 5 shows the distribution of suicides only among men, which makes it possible to observe that the median of cases for all years was always higher in May, but that this difference is significant (Kruskal-Wallis, p<0.01) only as of the year 2007.

**Fig 6:**
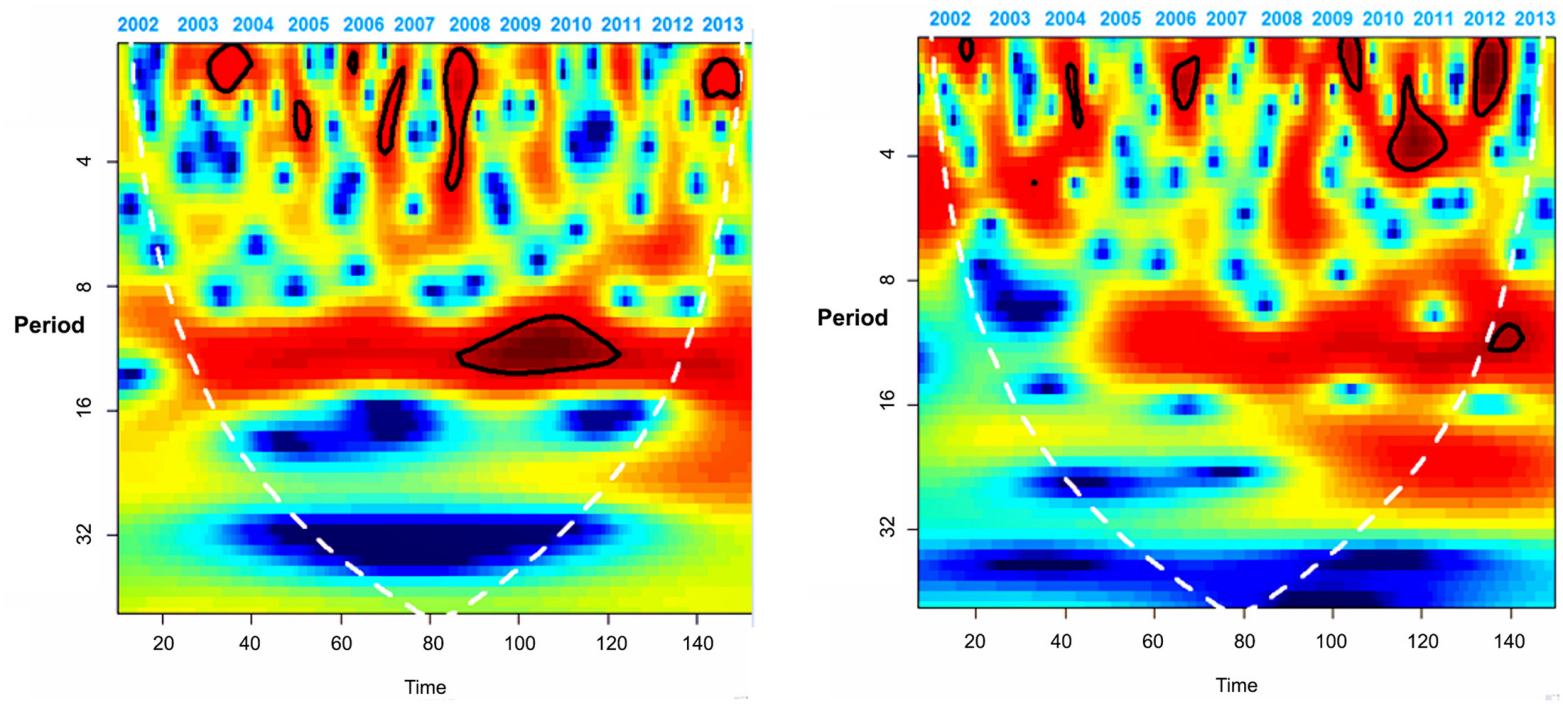
Wavelet Power Spectrum for the Time Series of Suicides for Men in the Urban (right) and Rural Areas (left). (Only as of 2002 due to a lack of data from previous years.)

**Fig 7:**
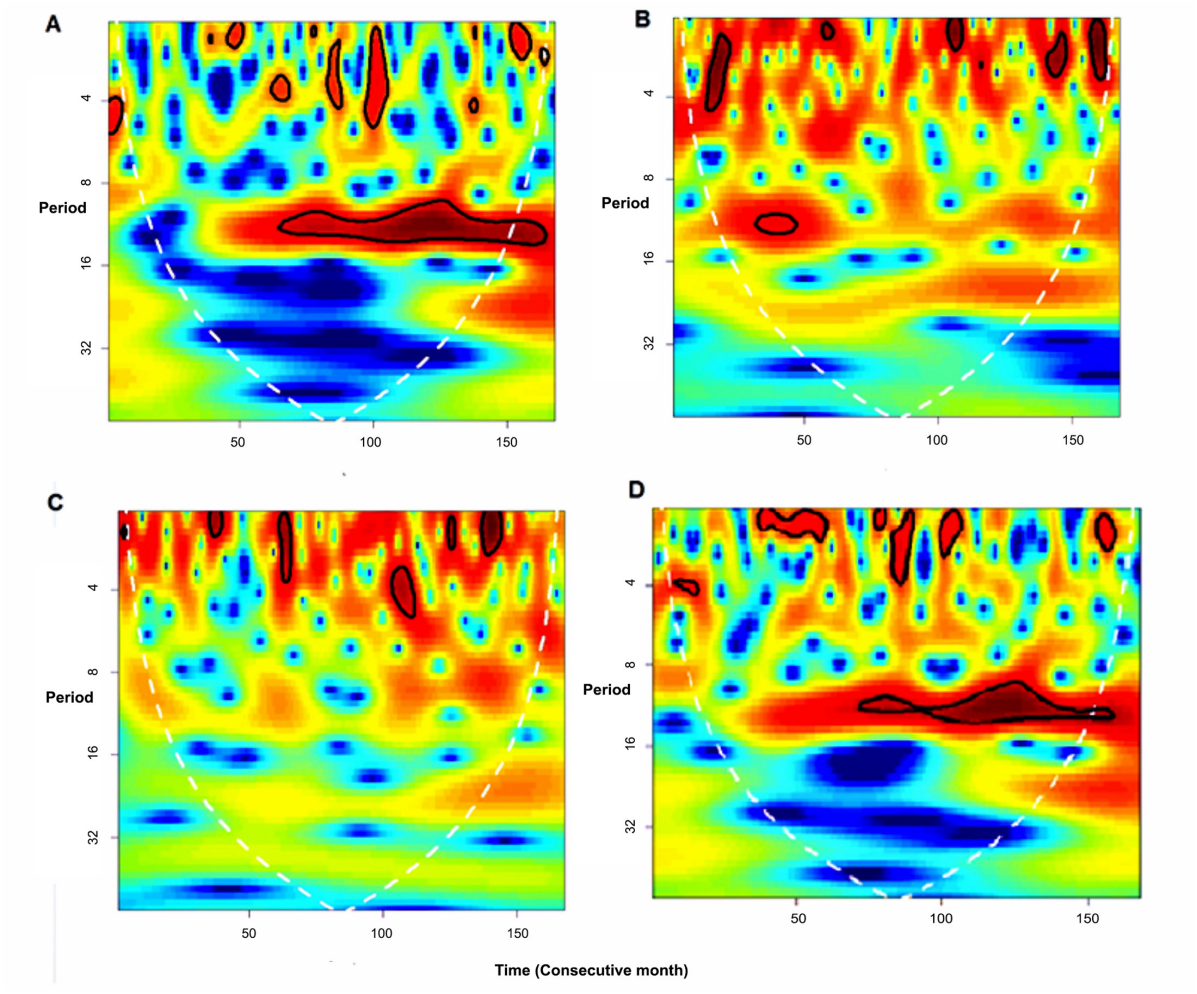
Wavelet Power Spectrum for the Time Series of Suicides per Mechanism, 2000–2013. *. * “A” represents suicide by hanging, asphyxiation or strangling, “B” by firearms and “C” by poisoning. “D” is all suicides without differentiating among the mechanisms used.

Lastly, when observing the differences between the time series in terms of urban/rural regions (Fig 6), this seasonality is only maintained for men in the urban area. The vertical red portion in the urban area indicates that this continues over time but is only statistically significant between 2008 and 2011. Nevertheless, this 12-month seasonal behavior was not observed in the series for men in the rural area, which is similar to the women. In terms of the behavior of the times series and their respective power spectra for women in urban and rural areas, no large differences were observed among all the women (Fig 4), and therefore these data are not shown. Fig 7 indicates the presence of a seasonal trend only for violent deaths, specifically those from asphyxia, hanging and strangling; although it should be noted that these represent 74% of the total.

**Fig 8:**
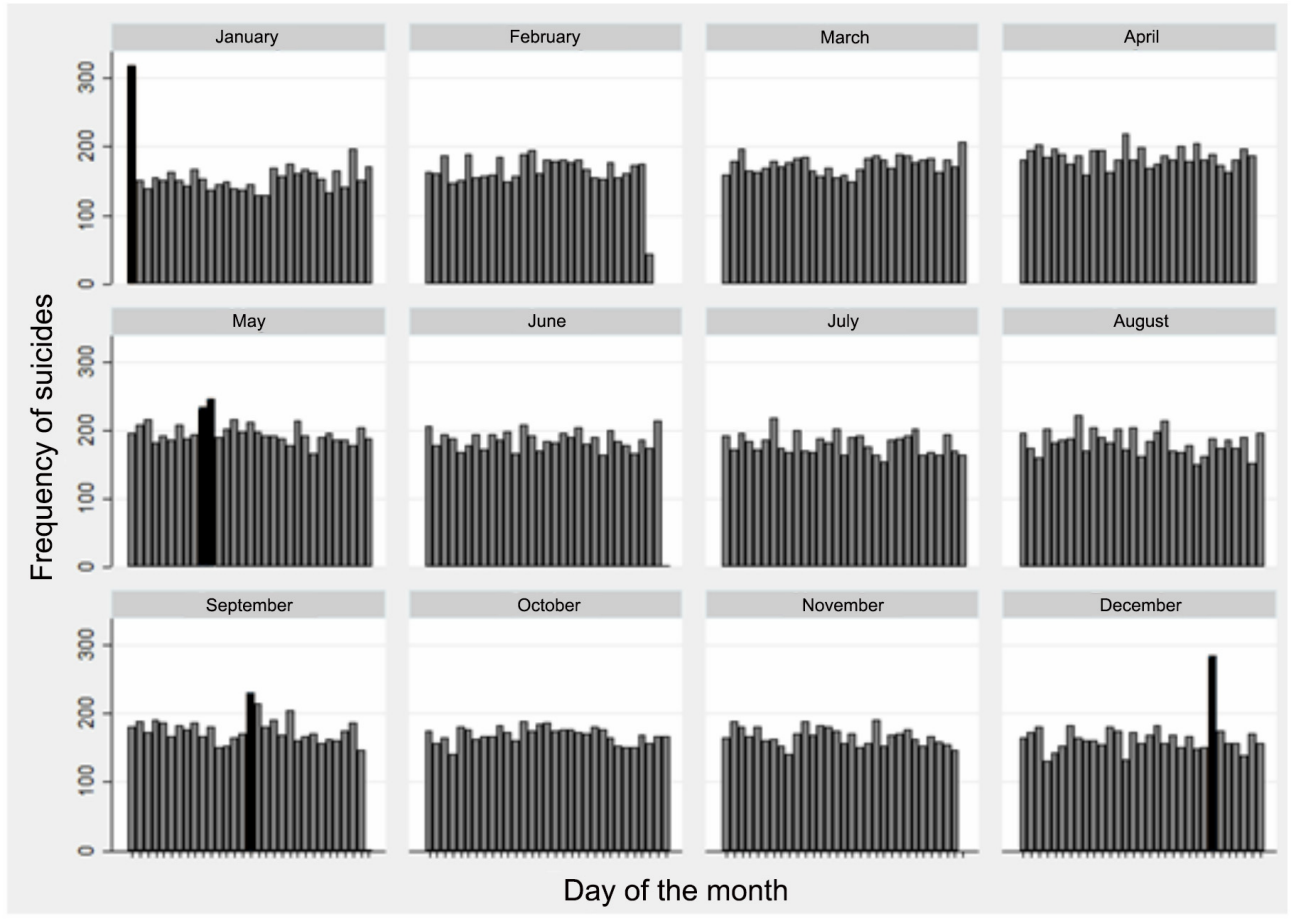
Distribution of Suicides in Mexico According to Day of the Year, 2000–2013*. * Differences are significant for each year and for all the years (p<0.05), using the Kruskal-Wallis Test, for each day versus any of the other days.

Lastly, Fig 8 shows the days of the year in the series that represent the largest number of suicides. A pattern was identified in which more suicides occurred on particular holidays: January 1 (New Year), May 10–11 (Mother’s Day), September 16 (Mexico Independence Day) and December 25 (Christmas) (*p*<0.05).

## Discussion

The main objective of this study was to explore the seasonal behavior of suicides in Mexico using a spectral decomposition of suicides registered between the years 2000 and 2013. Our results identified three components of seasonality related to deaths by suicide during this time period.

The first is seasonality related to the month of May. As mentioned previously, two types of explanations for this phenomenon have been offered: biological, which considers that elements related to the weather or the environment affect the functioning of neural and endocrine systems [7–10]; and sociological [11], which suggests that changes in social dynamics occurring during the seasons are responsible for this phenomenon. These explanations are not necessarily mutually exclusive and elements from both may contribute to the heterogeneity of the results, since the seasons involving risk could be related to a combination of social and weather-related factors that may vary by historical period or from region to region. Thus, for example, this pattern has been identified in some countries [18, 41, 42] while it has not been observed in others [43–45], or it has only been identified among men [41, 46], or only among women [47], while in other cases among both men and women [10]. Different results have also been observed in rural versus urban areas [10,48] and among some methods of suicide which may depend on the availability of the mechanisms over the course of a year[10,12,23,49]. This heterogeneity in the patterns is consistent with the findings from the present study which identified a seasonal pattern only among men in urban areas. Due to the limitations of the database, it is not possible to analyze whether the peak observed is larger for particular causes (grief, mental disorder, etc.) or if it is related to sex, rural areas or other variables, which is a topic of interest for future investigations.

The second component of the seasonality found by our study is an apparent increasing trend over the study period, especially as of the year 2007. In addition, the increase that occurred nearly every year in May only appears as of 2007, which is notable since that year coincides with the beginning of the “war on drugs” in Mexico, which has been associated with an increase in the levels of violence and deaths from homicide across the country [50]. One possible explanation for the larger number of suicides during this period could be an increase in everyday stressors ranging from the overall perception of a lack of safety to victimization. In addition, some deaths classified as suicides may correspond to unreported crimes, although this cannot be verified. This period is also marked by economic crisis, and although Mexico has been affected less than other countries this may have increased the perception of insecurity, unemployment and job instability, and decreased an overall sense of wellbeing [50].

The third component related to seasonality was the slight but consistent increase in suicides on the days following four holidays: Christmas, New Years, Mother’s Day and Mexico’s Independence Day. The increase in the number of suicides over these holidays has been reported previously [10,51] and is explained by “unfilled expectations” [52], according to which holidays create expectations of change and improvement which, when not fulfilled, leads to disappointment and suicide. Likewise, social stressors related to holidays may provoke suicides (while there is more contact with family or friends the sense of loneliness is greater when there is nobody with whom to spend the holidays). Nevertheless, findings related to this explanation also appear to be heterogeneous, and the differences in the effect of certain holidays as compared to others [53] are not always consistent with this theory of expectations.

Other explanations exist about the relationship between holidays and suicide. One is that more alcohol is consumed during these periods, and since this is associated with suicidal ideation and intent, and with suicide itself, alcohol consumption may be associated with suicide[1]. Another element of interest may be culturally specific, for example, the concept of a “good death” [54]. This refers to the belief by some people in rural areas in Mexico that dying during a religious holiday is particularly auspicious since beings such as saints, the Virgin Mary or God are in some way closer. Although the article cited refers to deaths from different diseases and only religious holidays, this is a topic which merits further investigation.

In summary, the three seasonal elements observed are likely due to a combination of explanations rather than a single reason. Since the peaks associated with holidays are consistent over the period, they appear to be related to circumstances that are common to those dates, some of which were mentioned above. Meanwhile, the causes of the seasonality of suicide *per se* seem to be less clear. As mentioned, it may be related to factors associated with the weather and its effect on neuro-physiological changes, or due to the social context or to differences in the availability of the methods for suicide during certain periods of the year. Furthermore, the seasonal pattern identified by the present study only corresponds to more violent methods, but since these represent the large majority of cases this finding may be due to its higher statistical power. Nevertheless, since the information available is not disaggregated by method or by geographic region, we are unable to draw conclusions of this type. This is another topic which should be continued to be investigated.

A complementary objective of this work is to explore the social distribution of cases of death from suicide in Mexico. Various theories indicate that social adversities is one of the most significant causes of suicide [55]. Many studies support this idea, finding that more suicides tend to occur in populations that are most disadvantaged in terms of schooling, income, employment and other indicators of social position [56]. Nevertheless, the effects vary among countries, regions, sex and other indicators. In low or medium income countries, both a lower education [57, 58] as well as a higher education [15,59, 60] have been reported to be associated with the risk of suicide. While our analysis does not include an indicator of income or socio-economic level, we found that most of the deaths from suicide during the study period occurred among those with a low level of schooling. The distribution of schooling for cases of death from suicide was at the elementary level or lower, while it was at the secondary level for the Mexican population 15 years of age or older in 2010 [61], suggesting an inverse relationship between educational level and suicide.

The rural area is another component associated with social position. In different regions around the world deaths from suicide are more common in rural areas. This may be related to poverty, unemployment or the isolation of the inhabitants in those areas. Other explanations have also been proposed, including toxicity from insecticides or the availability of certain means [60, 62, 63]. In our study, 22% of the suicides occurred in rural areas, a proportion which is close to that of the Mexican population living in those areas [64]. Thus, initially it seems that the rural region would not have an effect, although a more in-depth analysis would be needed in order to draw conclusions.

In terms of occupation, one of the most consistent findings reported in the literature is the relationship between unemployment and suicide [65]. According to our results, 3.5% of the males who completed suicide were unemployed. This is similar to the unemployment rate reported in Mexico for men 15 years of age and older (4.3%) [66] and, therefore, there appears to be no association. This topic also requires further analysis in order to determine conclusions, since the data available were not disaggregated and the classifications of work activities have changed. These findings should therefore be interpreted cautiously. Nevertheless, work and the stress associated with work has been reported to be a very relevant risk factor for suicide [67], a phenomenon which has not been studied in Mexico and which may be most important during economic crises such as those reported in various countries [68, 69].

Lastly, although suicide rates tend to be higher for men than for women, the differences vary in magnitude. Our study determined a rate of 4.6:1 in the number of suicides, which is higher than that reported by other studies [70, 71]. Various explanations have been suggested for the differences in completed suicides between males and females, including the lethality of the methods used by men [4,72], the tendency to adopt more self-destructive behaviors consistent with masculine gender roles in certain cultures [73], the means for suicide being more available to men and differences in patterns of substance use and in seeking out mental health care [1]. It is important to note that although we mention this information, given the nature of our data we cannot make inferences at the individual level.

In general, more studies are needed with other designs, information structures and analytical techniques in order to explore the association between social determinants and the occurrence of suicide in Mexico as well as the potential influence of these determinants on occurrence patterns over time. The discussions presented in the present work about the role of social factors are limited to hypotheses which need to be empirically developed and more extensively explored.

In particular, the results presented by this study regarding social factors need to be interpreted cautiously since they were derived only from the bivariate analyses and the social factors mentioned were only compared between men and women. Likewise, the wavelet analysis was performed only to evaluate differences in seasonality by sex, area of residence and method of suicide. It was not possible to include other covariables in the wavelet analysis because of the small number of cases in each group for each unit of time, and particularly for each combination of covariables. Their inclusion would also have gone beyond the main objective of the present analysis.

The limitations of this study were mainly the use of statistics from only one country and a particular time period. There was also a lack of information about history of mental health, disability, multi-morbidities, suicide methods, migration and life course, among others. These have been reported as conditions that could increase vulnerability to seasonal patterns [18, 49, 74], nevertheless their role in the occurrence and seasonality of suicide could unfortunately not be explored by the present study.

In actuality, the wavelet analysis may not be the most suitable method to establish multiple adjusted relations or to evaluate the independent influence of a variable on the occurrence and seasonality of an event. This technique only makes it possible to establish the existence of seasonality, whereas in order to explore the social or environmental determinants of this seasonality we would ideally need data that change over time. In this case, the wavelet analysis can be used to perform a “coherence analysis” [33], although these are typically only bivariate in order to observe the covariation between only two time series [33].

In addition, the number of suicides is very low at more local levels, such as the municipality and township, making it difficult to identify and statistically model patterns at those levels with any of the techniques available, including wavelet analyses. Thus, what is observed is the average behavior in the entire country rather than the potential heterogeneity at lower levels.

A possible underreporting or incorrect classification of deaths from suicide should also be considered [1]. This could result from deficiencies in recording the main cause of death on death certificates due to incorrectly filling out all the variables on the certificates or recording an unspecific cause regarding the intentionality of the injuries that caused the deaths [75, 76]. Indicators of an underreporting of suicides include the number of violent deaths that cannot be classified as either intentional or unintentional and the number of attempted suicides, which should be higher than the number of completed suicides [75]. Few studies have measured the underreporting of suicides. One in Canada reported this to be higher for women (17.5%) than for men (12%) [77]. Other studies have shown that underreporting may be significant and more frequent among groups for which suicide is culturally unacceptable, or it may be higher for women who die by drowning or poisoning, or in general from accidents or violent deaths for which intentionality cannot be established [60,75]. In the case of our data, a wrong classification is not likely given that 76% were suicides from asphyxiation, hanging or strangling. In addition, since each event was verified by a necropsy we do not consider underreporting to have had a substantial impact.

In spite of the limitations, to the best of our knowledge only one other study has reported on the relationship between climate and suicides in Mexico, and that was limited to one state in the country [78]. Considering the nature of the phenomenon, a strength of this work is the presentation of the wavelet analysis as a suitable option because of its usefulness in analyzing long time series, especially when mathematical properties change over time. It is also helpful for identifying different seasonal patterns that change over time, and therefore has been suggested as a suitable technique to explore the seasonality of suicide [79]. Therefore, the results from the present study contribute to the evidence showing that a spectral analysis is useful to study this phenomenon [25]. Furthermore, although initially the results can be generalized only for Mexico, it is reasonable to think that similar patterns, with the particularities in each country, could be found in comparable contexts.

In conclusion, suicide is a complex public health problem and potentially preventable. In order to design prevention programs, the phenomenon needs to be understood in-depth and its causes studied at the individual level as well as the intermediate (such as social and cultural resources provided by social networks and the locality) and macro levels (environment and social context). From an epidemiological perspective, one of the most interesting findings is the seasonal pattern of suicide, which indicates the importance of focusing prevention strategies on particular periods of the year. On the other hand, the heterogeneity of the seasonal pattern in relation to social elements is a topic that needs to be better understood, requiring an analsyis that takes into account other variables, their effects and interactions, and longer time periods[22]. Designs including psychological autopsies, cases and controls and attitude surveys may be useful to understand the complex relationships among the variables associated with suicide [80].
